# Circadian clock regulates the shape and content of dendritic spines in mouse barrel cortex

**DOI:** 10.1371/journal.pone.0225394

**Published:** 2019-11-15

**Authors:** Malgorzata Jasinska, Ewa Jasek-Gajda, Olga Woznicka, Grzegorz J. Lis, Elzbieta Pyza, Jan A. Litwin

**Affiliations:** 1 Department of Histology, Jagiellonian University Medical College, Krakow, Poland; 2 Department of Cell Biology and Imaging, Institute of Zoology and Biomedical Research, Jagiellonian University, Krakow, Poland; University of South Alabama, UNITED STATES

## Abstract

Circadian rhythmicity affects neuronal activity induced changes in the density of synaptic contacts and dendritic spines, the most common location of synapses, in mouse somatosensory cortex. In the present study we analyzed morphology of single- and double-synapse spines under light/dark (12:12) and constant darkness conditions. Using serial electron micrographs we examined the shape of spines (stubby, thin, mushroom) and their content (smooth endoplasmic reticulum, spine apparatus), because these features are related to the maturation and stabilization of spines. We observed significant diurnal and circadian changes in the shape of spines that are differentially regulated: single-synapse spines remain under circadian clock regulation, while changes of double-synapse spines are driven by light. The thin and mushroom single-synapse spines, regardless of their content, are more stable comparing with the stubby single-synapse spines that show the greatest diversity. All types of double-synapse spines demonstrate a similar level of stability. In light/dark regime, formation of new mushroom single-synapse spines occurs, while under constant darkness new stubby single-synapse spines are formed. There are no shape preferences for new double-synapse spines. Diurnal and circadian alterations also concern spine content: both light exposure and the clock influence translocation of smooth endoplasmic reticulum from dendritic shaft to the spine. The increasing number of mushroom single-synapse spines and the presence of only those mushroom double-synapse spines that contain spine apparatus in the light phase indicates that the exposure to light, a stress factor for nocturnal animals, promotes enlargement and maturation of spines to increase synaptic strength and to enhance the effectiveness of neurotransmission.

## Introduction

Maturation of a dendritic spine, associated with the level of its stability, is reflected in the shape of spine and/or in the content of spine organelles [1–5]. Precursors of dendritic spines, responsible for creating new synapses, filopodia [5–7] transform into stubby-shaped spines (stubby spines) [2,8]. Stubby spines are immature dendritic spines [2] and they do not have distinct neck and head compartments [9]. Stubby spines are transformed successively into thin-shaped spines (thin spines) and then into mushroom-shaped spines (mushroom spines) [2,10]. Thin and mushroom spines belong to the mature spines characterized by distinct heads and necks [2]. However, some disappearing mushroom spines pass through the stage of stubby shape [10]. It means that stubby spines may appear at the beginning as well as at the end of the spine lifetime. This process is more complex, because even mature spines could also arise *de novo* from dendritic shaft in response to neuronal activity and they might be formed irrespectively of the previously existing synapses in that area of the shaft [5,8,11].

Due to a small diameter of heads, thin spines belong to the category of small spines in contrast to large mushroom spines [12,13]. Mushroom spines contain more glutamate receptors than small ones [14,15]. The density of glutamate receptors in the spine membrane (mainly AMPA receptors in the neocortex) is positively correlated with the spine stability [15–17]. Indeed, thin spines have significantly fewer AMPA receptors than mushroom spines [14]. However, shapes of spines located close to each other (on the same dendritic segment) might be functionally more important than the shape of a single spine [18,19].

The available data also suggest a direct link between spine shape and its function. Thin spines (with narrow necks) show high mobility–they can be rapidly modified by neuronal activity changes (“write-enabled” spines) [20,21] and they are associated with learning new information (‘learning spines’) [2]. Mushroom spines (with large heads and relatively wide necks) are most stable spines (“write-protected” spines) [10,15,20–22] and they are considered to be involved in memory formation (‘memory spines’) [2].

The content of spines–membranous organelles: smooth endoplasmic reticulum (sER) and spine apparatus (SA), is also associated with the stability of spines. SA is built of a stack of sER cisternae separated by electron-dense material [23] containing synaptopodin, a SA-specific actin-binding protein [24]. SA is involved in the regulation of calcium storage and release [25–27], as well as in actin-dependent changes in the spine cytoskeleton, including stabilization of actin filaments and support of actin polymerization [28,29], i.e. in processes participating in synaptic plasticity (for review see: [30]). The absence of synaptopodin, which results in the lack of SA, causes long-term potentiation deficits and impaired learning [24,30]. Moreover, SA/synaptopodin in cooperation with polyribosomes present in spines, participate in local protein synthesis [31,32], being responsible for its induction and contributing to post-translational modifications of proteins [33]. The presence of SA in dendritic spine seems to result in spine head enlargement, increased accumulation of postsynaptic AMPA receptors and enhancement of synaptic strength [26,30,34].

Spines containing SA are most stable and sER/SA-free spines are most transient ones [4,15,35]. Mushroom spines contain SA more often than the other spine types and thin spines usually are sER/SA-free [12,36]. It confirms the existence of a continuum of spine maturation and stability also reflected by a sequence of spine content (no membranous structures–sER–SA), with small and large spines representing the two extremes.

The majority of earlier observations regarding dendritic spines have concerned the experience-dependent plasticity or neuropathological processes, and there are no studies investigating dendritic spine morphology in the cerebral cortex under different light conditions during 24 h. Some authors reported spine modifications associated with sleep [37,38] but as far as the clock-induced changes are concerned, it is significant to note that the rest phase of mice is not synonymous with sleep and during the activity phase the animals are not in permanent wakefulness (arousal) [39]. A study of Ikeda et al. [40] presented changes in the number of hippocampal spines, categorized according to the size of their heads, under light/dark regime, but it did not include a similar analysis in constant darkness which would allow to draw conclusions concerning the influence of the biological clock on the observed changes.

We have previously found that the circadian clock differentially regulates the number of single-synapse spines (spines with a single excitatory synapse) and double-synapse spines (spines with two different synapses–one excitatory and the other one inhibitory) in mouse somatosensory cortex [41]. Although single-synapse spines increase in the number during the day (light/dark 12 : 12 –LD12:12)/subjective day (constant darkness–DD), double-synapse spines are more numerous during the night (LD12:12)/subjective night (DD). Interestingly, the inhibitory synapses are strongly regulated by the clock, while excitatory synapses were more driven by the light exposure in LD 12:12 conditions [41].

The changes in the density of dendritic spines are often accompanied by their morphological modifications reflecting stability, maturation level and functionality of the spines. Therefore, in the present study we examined morphological changes of the spines including their shape and content in LD 12:12 and DD conditions. We observed diurnal and circadian rhythmicity in morphological modifications of different spine types.

## Materials and methods

### Animals

Sixteen male C57BL6/cmdb mice aged 5–6 weeks (Center of Experimental Medicine, Medical University of Bialystok, strain imported from The Jackson Laboratory) were used in the study. The experiments were carried out in accordance with the Council Directive 2010/63EU of the European Parliament and the Council of 22 September 2010 on the protection of animals used for scientific purposes and approved by the Animal Care and Use Committee of the Jagiellonian University in Krakow, Poland.

### Analysis of locomotor activity

All animals were kept for 10–14 days at LD 12:12 (light 60 lx), 25°C and 50% humidity to accommodate to standard light conditions. Next, mice were divided into LD (n = 8) and DD (n = 8) groups. The LD mice were kept for the next 10–14 days under LD 12:12 and DD mice–under constant darkness. The animals were fed a standard diet and water *ad libitum*.

From the beginning of experiments mice were kept singly in cages with free access to a running wheel coupled with a 16-channel electromagnetic pulse counter (MIKI 1; Autel; Krakow, Poland). The number of wheel rotations per minute was continuously recorded, transferred to PC computer by RS232 interface and saved using RealTerm software (RealTerm: Serial/TCP Terminal 2.0.0.70; realterm.sf.net). The obtained data were analysed using NIH ImageJ ActogramJ software (http://rsb.info.nih.gov/ij/).

**Fig 1:**
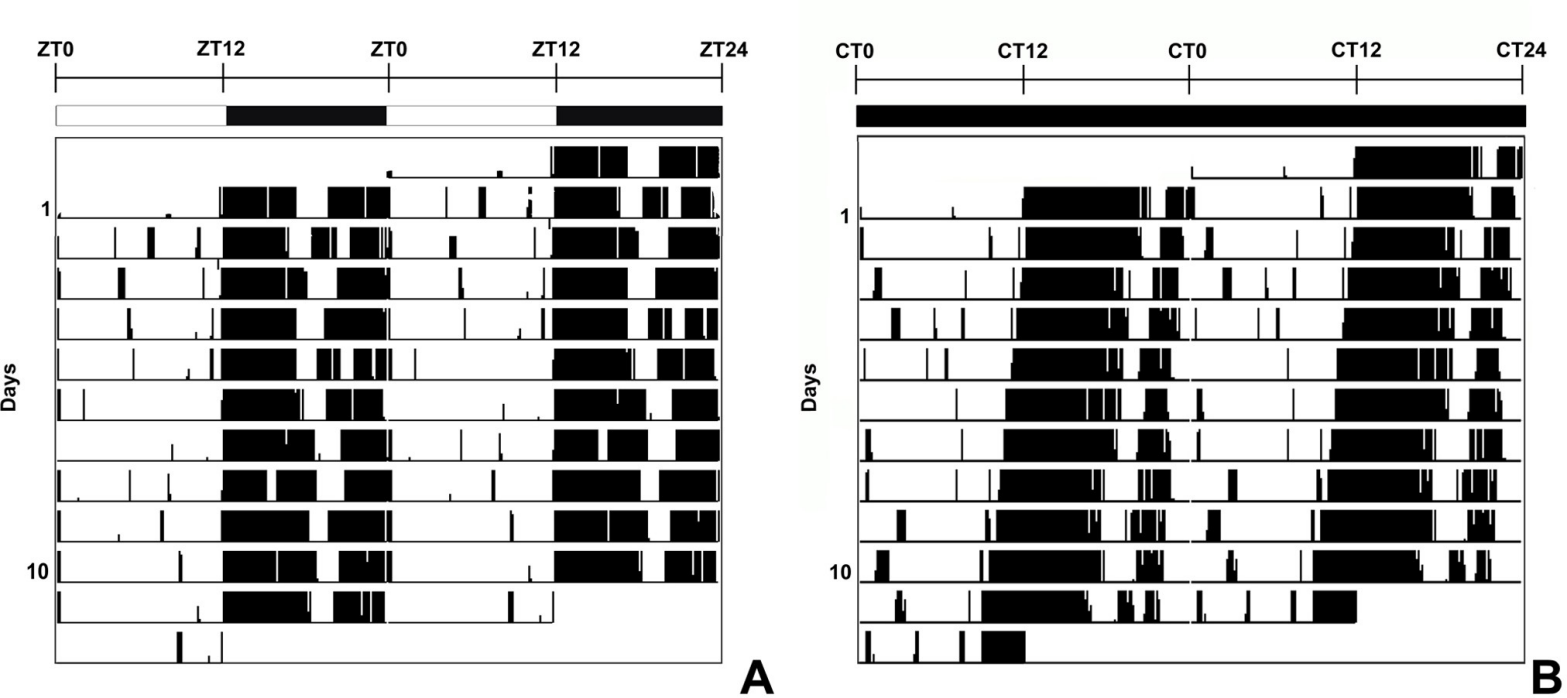
Representative double-plotted actograms of the running-wheel locomotor activity of mice. Animals showed locomotor rhythmicity under light/dark 12:12 (A) and constant darkness conditions (B). ZT–Zeitgeber time, CT–circadian time.

All mice showed locomotor rhythmicity under LD conditions (Fig 1) and were used for further experiments. The intensity of locomotor activity within the 24h period did not differ between animals kept in LD and DD conditions [41]. Eight mice were sacrificed 2 h after the beginning of the light phase of LD 12:12 or the subjective day of DD (n = 4 per LD REST and DD REST) and 8 mice were sacrificed 2 h after the beginning of the dark phase (LD 12:12) or the subjective night (DD; n = 4 per LD ACTIVE and DD ACTIVE). The above-mentioned time points—2 hours after the beginning of the rest or activity phases were chosen to make sure that the results reflect the effect of the analyzed phase and are not a consequence of the earlier phase. Moreover, in our previous study we found clear differences in the density of synapses and spines between the same two time points [41]. Hence, their repetition gives us the opportunity to check whether the quantitative changes observed in the earlier study are accompanied by morphological changes.

### Transmission electron microscopy

Mice were anesthetized with Morbital (25–30 mg/kg b.w.; Biowet, Pulawy, Poland) and perfused through the heart with 20 ml of rinse buffer (0.2% glutaraldehyde and 2% paraformaldehyde in 0.1 M phosphate buffer, pH 7.4) followed by 100–150 ml of fixative (2.5% glutaraldehyde and 2% paraformaldehyde in 0.1 M phosphate buffer, pH 7.4). Immediately after perfusion, the brains were removed and left in the same fixative for 24 h at 4°C.

Next, after washing in 0.1 M phosphate buffer (pH 7.4), 60 μm tangential vibratome sections were cut from the barrel cortex region and examined under a stereomicroscope (Nikon Optiphot, Japan). Only sections containing the barrel field cortex were collected for further processing. The sections were washed in 0.1 M cacodylate buffer (pH 7.4), postfixed twice with 1% osmium tetroxide in 0.1 M cacodylate buffer, pH 7.4 (two times for 1 h, the first change containing 1.5% potassium ferrocyanide), washed in 70% ethanol containing 1% uranyl acetate, and after dehydration in graded series of ethanol, embedded in Epon (Polysciences, USA) between two silicon-coated glass slides.

The region of B2 barrel was identified according to the procedure described previously [42]. The embedded slices were trimmed into blocks and series of 10–15 successive ultrathin sections (65–75 nm thick) were cut from each sample. The sections were collected on formvar-coated copper-palladium slot grids and contrasted with 1% lead citrate.

Synapses and spines were defined according to Knott et al. [43]. Synapses were characterized by two apposed, thickened membranes with an identifiable cleft in between and a presynaptic component containing vesicles. The assessment of synapse type (excitatory or inhibitory) was based on the symmetry of synaptic membranes (asymmetric/symmetric) and on the appearance of synaptic vesicles (round/ovoidal). The accordance of excitatory and inhibitory synapse morphology with glutamate and GABA synaptic markers was previously verified by immunocytochemistry [42]. Dendritic spines (seen in the several adjacent micrographs) were identified by their small size relative to the larger dendrite, the unique features of their profiles and the absence of microtubules and mitochondria.

For examination of dendritic spine morphology, from each mouse 3–5 series of electron micrographs (10–15 serial micrographs each) of the B2 barrel central area in which cell bodies are sparse, were taken at 30 K under a JEOL JEM 2100 transmission electron microscope (JEOL, Japan). The micrographs were aligned using Adobe Photoshop CS software and stacks of serial images were prepared.

### Morphological analysis of single- and double-synapse spines

Only the spines fully located within each stack of images were selected for examination. Images of 171 single-synapse spines and 89 double-synapse spines from both groups were chosen for morphological measurements and 3D reconstruction (single synapse-spines–LD REST: 42, LD ACTIVE: 37, DD REST: 51, DD ACTIVE: 41; double synapse-spines–LD REST: 18, LD ACTIVE: 25, DD REST: 20, DD ACTIVE: 26; 16 ± 3 spines/animal).

**Fig 2:**
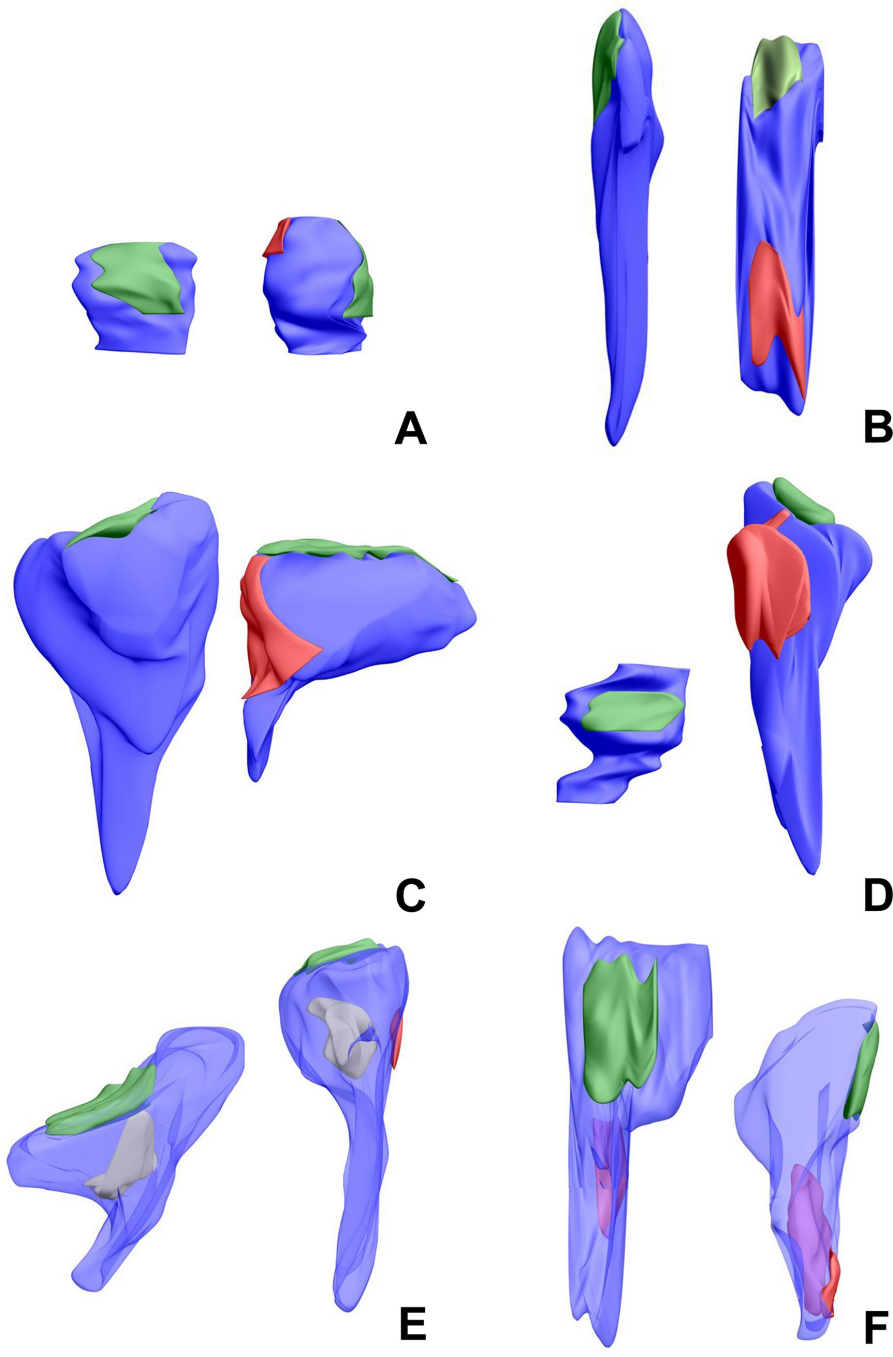
3D reconstructions of single- and double-synapse spines. Pictures show different spine shapes: stubby (A), thin (B), mushroom (C), intermediate (D) and different contents: sER (white, E) and SA (red, F). Excitatory and inhibitory synapses are marked in green and red, respectively.

The shapes of spines were defined according to [44] and divided into three categories: with the use of the following criteria: stubby-shaped spines (l ≈ d_n_, where l is the length of spine and d_n_ is the diameter of spine neck), thin-shaped spines (l ≥ 3 x d_n_ and d_h_ ≈ d_n_, where d_h_ is the diameter of spine head), and mushroom-shaped spines (d_h_ ≥ 2.5 x d_n_; Fig 2A–2C; [44,45]). Some spines showed intermediate features (Fig 2D). This group included spines with shapes intermediate between the basic categories: stubby/thin (l = 2–3 x d_n_ and d_h_ ≈ d_n_) and thin/mushroom spines (l ≥ 3 x d_n_ and 2.5 x d_n_ >d_h_ > 1.5 x d_n_). Generally, intermediate spines constituted about 30% and 16% of single-synapse and double-synapse spines, respectively. Because of large number of intermediate spines, they were treated as separate spine shapes in addition to stubby, thin and mushroom spines. Using serial electron micrographs of spines, their content was identified and according to that criterion spines were divided into three types: spines containing smooth ER (sER; Fig 2E), spines containing spine apparatus (SA; Fig 2F) and spines without these structures (sER-free). In case of intermediate spines, separate statistical analysis of their content in the two subgroups was impossible because of high variability of parameters within the subgroups and/or the absence of some spine variants, therefore stubby/thin and thin/mushroom spines were combined into one group of intermediate spines for this analysis.

### Statistical analysis

All measurements were performed using NIH ImageJ software (Analyze-Measure, Cell Counter Plugin; http://rsb.info.nih.gov/ij/).

The counting was done blind–the observer did not know whether the micrographs were taken from LD or DD and ACTIVE or REST groups.

All data were analysed using GraphPad Prism 5.01 software (GraphPad Software Inc., La Jolla, CA, USA).

Differences in the sampling area across the experimental groups were analyzed by Kolmogorov-Smirnov normality test and homogeneity Bartlett’s test for equal variances, followed by one-way ANOVA test with post hoc Tukey’s test. To compare the combined effect of light conditions and spine content on the morphological data across the experimental groups, two-way ANOVA with post hoc Bonferroni test preceded by Kolmogorov-Smirnov normality test was used. In the Results section and in figures, data are presented as means ± SD and means ± SEM, respectively.

## Results

### Sampling areas

Dendritic spines were selected from samples with the following total tissue volumes: LD REST group– 554.64 ± 6.93 μm^3^ (mean volume per animal 138.66 ± 4.16 μm^3^), LD ACTIVE group– 567.85 ± 7.10 μm^3^ (mean volume per animal 141.96 ± 4.26 μm^3^), DD REST group– 541.44 ± 6.77 μm^3^ (mean volume per animal 135.36 ± 4.06 μm^3^), DD ACTIVE group– 607.47 ± 7.59 μm^3^ (mean volume per animal 151.87 ± 4.56 μm^3^). The volume of samples did not significantly differ across the groups (F(3, 12) = 0.189, p = 0.901, one-way ANOVA).

### Shape of spines

#### Single-synapse spines

**Fig 3:**
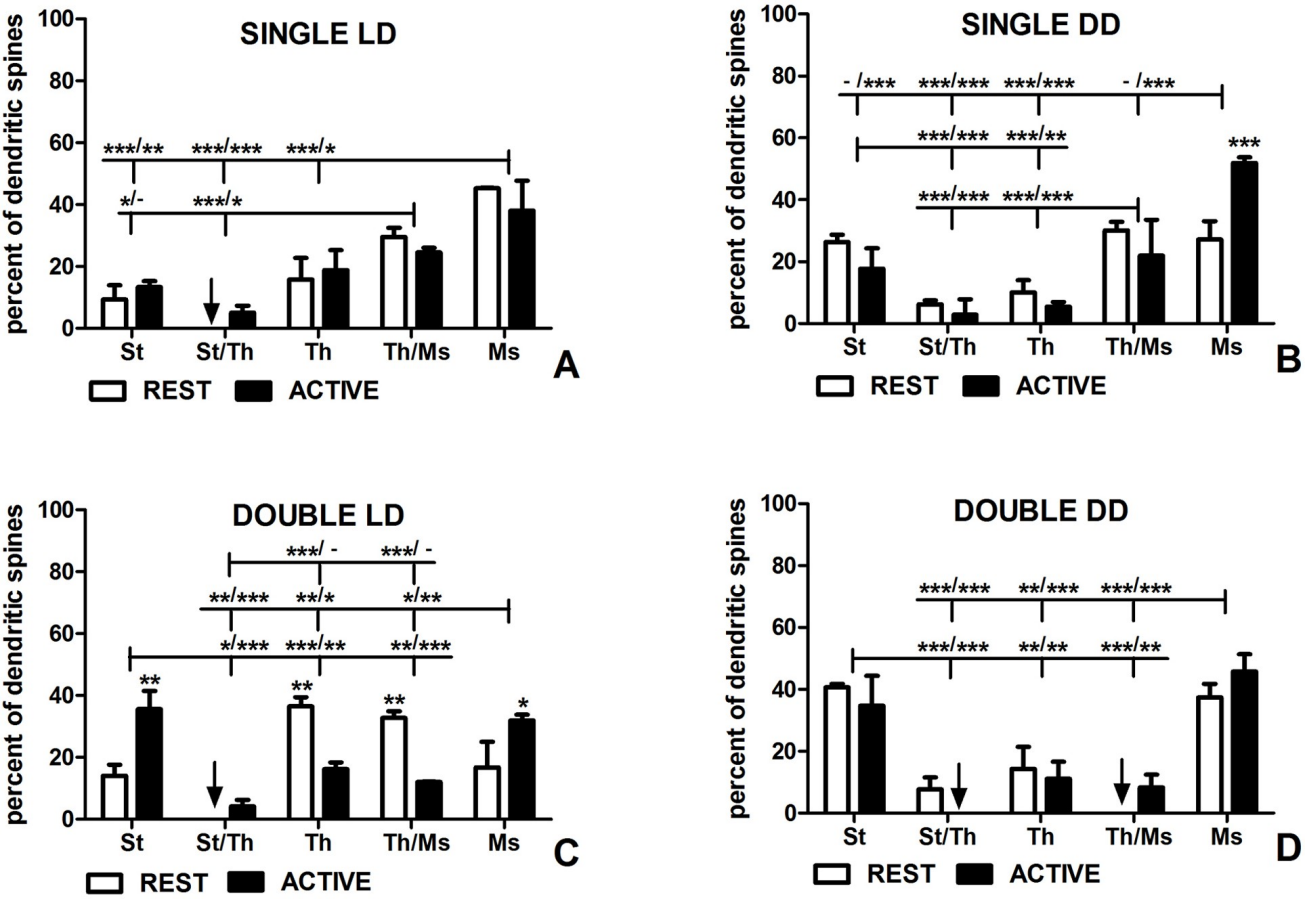
Shapes of dendritic spines in groups REST and ACTIVE under LD and DD conditions. **Shape changes of single-synapse and double-synapse spines are driven by circadian clock and light, respectively.** (A) Shapes of single-synapse spines under LD conditions. (B) Shapes of single-synapse spines under DD conditions. (C) Shapes of double-synapse spines under LD conditions. (D) Shapes of double-synapse spines under DD conditions. The graphs show means ± SEM (two-way ANOVA; *** p < 0.001, ** p < 0.01, * p < 0.05). Arrows indicate the absence of spines. The asterisks above the bars indicate significant differences in the percentage of spines of the same shape between activity phases (REST vs ACTIVE), while the asterisks above lines show significant differences between spines of different shapes for the corresponding REST / ACTIVE phase. St–stubby; St/Th–stubby/thin; Th–thin; Th/Ms–thin/mushroom; Ms–mushroom.

In the LD group, stubby/thin single-synapse spines were absent in the subgroup REST (Fig 3A, S1 Table). Mushroom single-synapse spines were more numerous as compared to thin single-synapse spines (REST–threefold, t = 4.400, p < 0.001; ACTIVE–twice, t = 2.848, p < 0.05), stubby/thin single-synapse spines (REST–stubby/thin: absent, t = 6.748, p < 0.001; ACTIVE–sevenfold, t = 4.896, p < 0.001) and stubby single-synapse spines (REST–fivefold, t = 5.349, p < 0.001; ACTIVE–twice, t = 3.675, p < 0.01; F(4, 30) = 20.06, p < 0.0001, two-way ANOVA; Figs 2 and 3A). Thin/mushroom single-synapse spines were more frequent than stubby/thin single synapse spines irrespective of the activity phases of animals (REST–t = 4.400, p < 0.001; ACTIVE–fourfold, t = 2.892, p < 0.05; F(4, 30) = 20.06, p < 0.0001, two-way ANOVA; Fig 3A). Moreover, thin/mushroom single-synapse spines were threefold more numerous when compared with stubby single-synapse spines in the subgroup REST (t = 3.001, p < 0.05; F(4, 30) = 20.06, p < 0.0001, two-way ANOVA; Fig 3A). However, there were no differences in single-synapse spines of the same shape between the subgroups REST and ACTIVE (F(4, 30) = 0.72, p = 0.585, two-way ANOVA; Fig 3A).

In the DD group, mushroom shape predominated in single-synapse spines in the subgroup ACTIVE and mushroom single-synapse spines were twice as numerous as thin/mushroom single-synapse spines (t = 8.093, p < 0.001), nine times more numerous than thin single-synapse spines (t = 12.550, p < 0.001), seventeen times more frequent than stubby/thin single-synapse spines (t = 13.250, p < 0.001), and almost threefold more frequent than stubby single-synapse spines (t = 9.242, p < 0.001; F(4, 30) = 59.13, p < 0.0001, two-way ANOVA; Fig 3B). Additionally, stubby and thin/mushroom single-synapse spines were more numerous comparing with stubby/thin and thin single-synapse spines in the subgroup ACTIVE (stubby vs stubby/thin: sixfold, t = 4.007, p < 0.001; stubby vs thin: threefold, t = 3.304, p < 0.01; thin/mushroom vs stubby/thin: sevenfold, t = 5.156, p < 0.001; thin/mushroom vs thin: almost fourfold, t = 4.453, p < 0.001; F(4, 30) = 59.13, p < 0.0001, two-way ANOVA; Fig 3B). In the subgroup REST, mushroom, thin/mushroom and stubby single-synapse spines were similarly numerous and fourfold more frequent than the other two types of single-synapse spines (mushroom vs thin: t = 4.590, p < 0.001; mushroom vs stubby/thin: t = 5.663, p < 0.001; thin/mushroom vs thin: t = 5.406, p < 0.001; thin/mushroom vs stubby/thin: t = 6.480, p < 0.001; stubby vs thin: t = 4.376, p < 0.001; stubby vs stubby/thin: t = 5.450, p < 0.001; F(4, 30) = 59.13, p < 0.0001, two-way ANOVA; Fig 3B). Generally, stubby/thin and thin spines were the rarest single-synapse spines regardless of the activity phase of animals. Moreover, mushroom single-synapse spines were almost twice more frequent in the subgroup ACTIVE when compared to REST (t = 6.696, p < 0.001; F(4, 30) = 14.39, p < 0.0001, two-way ANOVA; Fig 3B).

#### Double-synapse spines

In the LD group, stubby/thin double-synapse spines were absent in the subgroup REST (Fig 3C, S2 Table). Thin and thin/mushroom spines were the predominant populations of double-synapse spines (each group twice as numerous comparing with stubby and mushroom spines) in the subgroup REST (thin vs stubby: t = 4.204, p < 0.001; thin vs mushroom: t = 3.716, p < 0.01; thin vs stubby/thin: t = 6.844, p < 0.001; thin/mushroom vs stubby: t = 3.510, p < 0.01; thin/mushroom vs mushroom: t = 3.022, p < 0.05; thin/mushroom vs stubby/thin: t = 6.150, p < 0.001; F(4,30) = 14.40, p < 0.0001, two-way ANOVA), and they were also more frequent when compared with the subgroup ACTIVE (thin: twice, t = 3.805, p < 0.01; thin/mushroom: twice, t = 3.891, p < 0.01; F(4,30) = 13.71, p < 0.0001, two-way ANOVA; Figs 2 and 3C). Significant differences were also found between stubby as well as mushroom and stubby/thin double-synapse spines in the subgroup REST (stubby: t = 2.640, p < 0.05; mushroom: t = 3.127, p < 0.01; F(4,30) = 14.40, p < 0.0001, two-way ANOVA; Fig 3C). In the subgroup ACTIVE, stubby and mushroom double-synapse spines were more frequent comparing with stubby/thin, thin, and thin/mushroom double-synapse spines (stubby vs stubby/thin: eightfold, t = 5.906, p < 0.001; stubby vs thin: twice, t = 3.649, p < 0.001; stubby vs thin/mushroom: almost threefold, t = 4.429, p < 0.001; mushroom vs stubby/thin: sevenfold, t = 5.210, p < 0.001; mushroom vs thin: almost twice, t = 2.953, p < 0.05; mushroom vs thin/mushroom: twice, t = 3.733, p < 0.01; F(4,30) = 14.40, p < 0.0001, two-way ANOVA; Fig 3C). The percentage of stubby and mushroom double-synapse spines in the subgroup ACTIVE were twice as high as in the subgroup REST (stubby: t = 4.049, p < 0.01; mushroom: t = 2.865, p < 0.05; F(4,30) = 13.71, p < 0.0001, two-way ANOVA; Fig 3C).

In the DD group, stubby/thin double-synapse spines as well as thin/mushroom double-synapse spines were absent in the subgroups ACTIVE and REST, respectively (Fig 3D). Stubby double-synapse spines were twice and threefold as numerous as thin double-synapse spines in the subgroups REST (t = 3.671, p < 0.01) and ACTIVE, respectively (t = 3.286, p < 0.01; F(4,30) = 26.05, p < 0.0001, two-way ANOVA; Fig 3D). They were fivefold more frequent when compared with stubby/thin double-synapse spines in the subgroup REST (t = 4.589, p < 0.001) and fourfold more numerous comparing with thin/mushroom in the subgroup ACTIVE (t = 3.673, p < 0.01; F(4,30) = 26.05, p < 0.0001, two-way ANOVA; Fig 3D). Moreover, there were significant differences between stubby and stubby/thin double-synapse spines in the subgroup ACTIVE (t = 4.833; p < 0.001) as well as thin/mushroom double-synapse spines in the subgroup REST (t = 5.660, p < 0.001; F(4,30) = 26.05, p < 0.0001, two-way ANOVA; Fig 3D). Similarly, mushroom double-synapse spines were twice and fourfold as numerous as thin double-synapse spines in the subgroups REST (t = 3.211, p < 0.01) and ACTIVE, respectively (t = 4.833, p < 0.001), fourfold more frequent than stubby/thin double-synapse spines in the subgroup REST (t = 4.130, p < 0.001) and fivefold more numerous than thin/mushroom double-synapse spines in the subgroup ACTIVE (t = 5.220, p < 0.001; F(4,30) = 26.05, p < 0.0001, two-way ANOVA; Fig 3D). Significant differences were also observed between mushroom double-synapse spines and stubby/thin double-synapse spines in the subgroup ACTIVE (t = 6.379, p < 0.01) as well as thin/mushroom double-synapse spines in the subgroup REST (t = 5.200, p < 0.001; F(4,30) = 26.05, p < 0.0001, two-way ANOVA; Fig 3D). There were no differences in the percentage of spines, irrespective of their shape, between activity phases (F(4,30) = 1.190, p = 0.335, two-way ANOVA; Fig 3D).

### Shape to content relationship in single-synapse spines

#### Stubby spines

**Fig 4:**
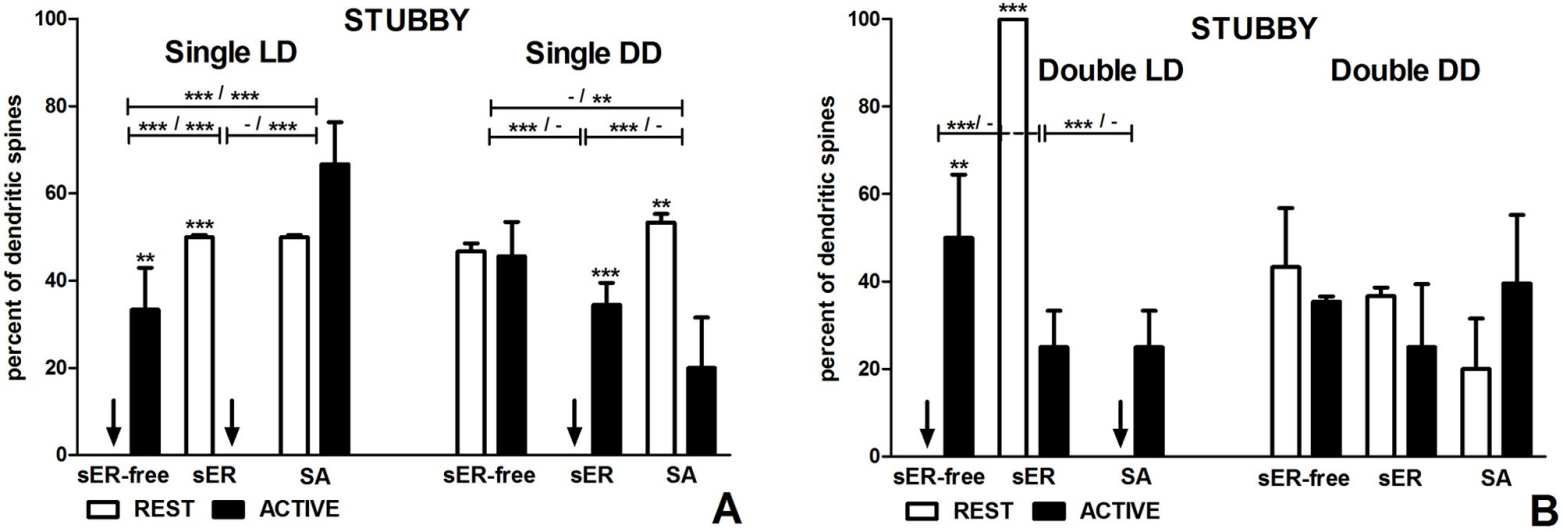
Content of stubby spines in groups REST and ACTIVE under LD and DD conditions. **Content changes in single-synapse spines are driven by both light and circadian clock, changes in double-synapse spines are driven exclusively by light.** (A) Stubby single-synapse spines. (B) Stubby double-synapse spines. The graphs show means ± SEM (two-way ANOVA; *** p < 0.001, ** p < 0.01, * p < 0.05). Arrows indicate the absence of spines. The asterisks above the bars indicate significant differences in the percentage of spines with the same content between activity phases (REST vs ACTIVE), while the asterisks above lines show significant differences between spines with different contents for the corresponding REST / ACTIVE phase. sER-free–no membraneous structures; sER–smooth endoplasmic reticulum; SA–spine apparatus.

In the LD group, there were no stubby sER-free single-synapse spines and spines containing sER in the subgroups REST and ACTIVE, respectively, and it was associated with significant changes of spine fractions between different activity phases (sER-free: t = 4.138, p < 0.01; sER: t = 6.207, p < 0.001; F(2,15) = 29.97, p < 0.0001, two-way ANOVA; Figs 2 and 4A). Consequently, stubby sER-free single-synapse spines were significantly more numerous than stubby single-synapse spines containing sER in the subgroup ACTIVE (t = 4.469, p < 0.001), and, conversely, stubby single-synapse spines containing sER as well as containing SA were more frequent comparing with stubby sER-free single-synapse spines in the subgroup REST (sER: t = 5.807, p < 0.001; SA: t = 5.807, p < 0.001; F(2,15) = 29.97, p < 0.0001, two-way ANOVA; Fig 4A). The most numerous stubby single-synapse spines were spines containing SA in the subgroup ACTIVE (by twofold comparing with sER-free spines; sER-free: t = 4.471, p < 0.001; sER: t = 8.940, p < 0.001; F(2,15) = 29.97, p < 0.0001, two-way ANOVA), however, these spines did not show differences in the percentage between activity phases (t = 2.070, p > 0.05; Fig 4A).

In the DD group, there were no differences in the percentage of stubby sER-free single-synapse spines between the subgroups REST and ACTIVE (t = 0.147, p > 0.05; Fig 4A). Such differences were observed in case of stubby single-synapse spines containing sER which were absent in the subgroup REST (t = 4.562, p < 0.001) and stubby single-synapse spines containing SA which were twice more frequent in the subgroup REST than ACTIVE (t = 4.415, p < 0.01; F(2,18) = 20.16, p < 0.0001, two-way ANOVA; Fig 4A). The absence of stubby single-synapse spines containing sER caused the appearance of significant differences when these spines were compared with stubby sER-free single-synapse spines and stubby single-synapse spines containing SA in the subgroup REST (sER-free: t = 6.182, p < 0.001; SA: t = 7.065, p < 0.001; F(2,18) = 15.24, p = 0.0001, two-way ANOVA; Fig 4A). Additionally, stubby sER-free single-synapse spines were twice as numerous as stubby single-synapse spines containing SA in the subgroup ACTIVE (t = 3.386, p < 0.01; F(2,18) = 15.24, p = 0.0001, two-way ANOVA; Fig 4A).

#### Thin spines

**Fig 5:**
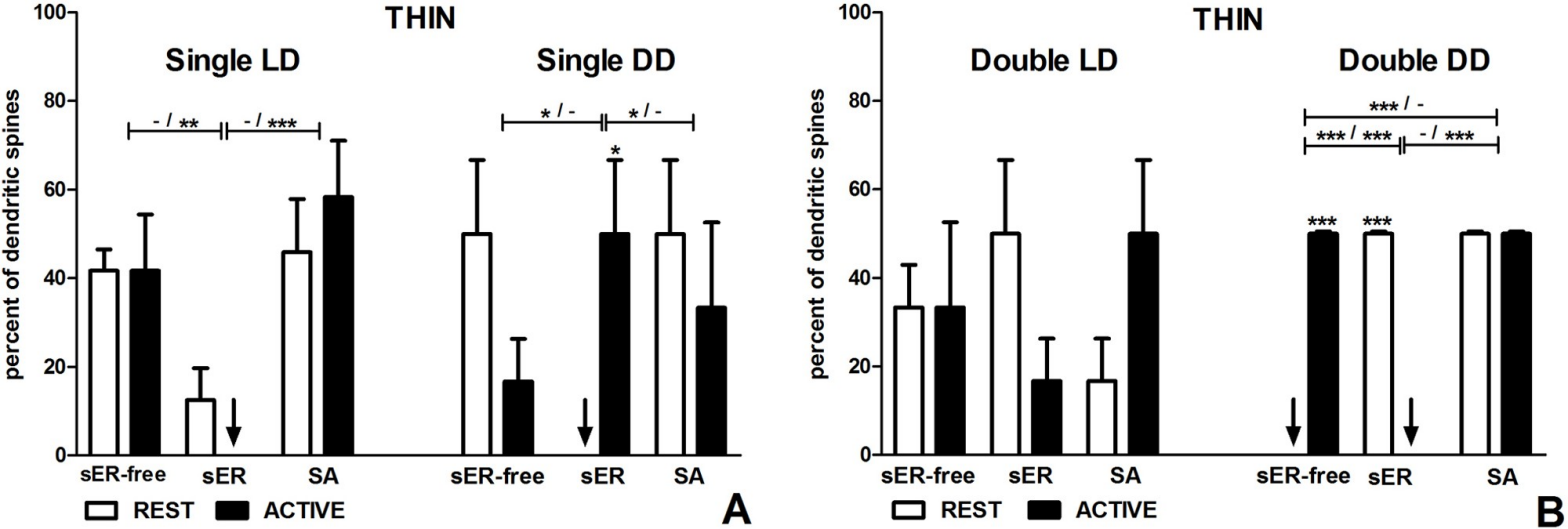
Content of thin spines in groups REST and ACTIVE under LD and DD conditions. **Content changes in single-synapse spines are driven mainly by circadian clock, changes in double-synapse spines are driven by both, circadian clock and light.** (A) Thin single-synapse spines. (B) Thin double-synapse spines. The graphs show means ± SEM (two-way ANOVA; *** p < 0.001, ** p < 0.01, * p < 0.05). Arrows indicate the absence of spines. The asterisks above the bars indicate significant differences in the percentage of spines with the same content between activity phases (REST vs ACTIVE), while the asterisks above lines show significant differences between spines with different contents for the corresponding REST / ACTIVE phase. sER-free–no membranous structures; sER–smooth endoplasmic reticulum; SA–spine apparatus.

In the LD group, there were no differences in the percentage of thin single-synapse spines between the subgroups REST and ACTIVE regardless of the spine content (F(2,15) = 0.95, p = 0.410, two-way ANOVA; Figs 2 and 5A). However, thin sER-free single-synapse spines as well as thin single-synapse spines containing SA were significantly more numerous than thin single-synapse spines containing sER in the subgroup ACTIVE (sER-free: t = 3.506, p < 0.01; SA: t = 4.908, p < 0.001; F(2,15) = 14.01, p = 0.0004, two-way ANOVA; Fig 5A). It was due to the absence of thin single-synapse spines containing sER in the activity phase (Fig 5A).

In the DD group, no single-synapse spines containing sER were found in the subgroup REST, therefore thin sER-free single-synapse spines as well as thin single-synapse spines containing SA were significantly more numerous in that subgroup (sER-free: t = 2.777, p < 0.05; SA: t = 2.777, p < 0.05; F(2,18) = 5.99, p = 0.010, two-way ANOVA; Fig 5A). Consequently, there was a statistically significant difference in the percentage of thin single-synapse spines containing sER between the subgroups REST and ACTIVE (t = 2.777, p < 0.05; F(2,18) = 5.99, p = 0.010, two-way ANOVA; Fig 5A). However, there were no differences between thin single-synapse spines with different content in the subgroup ACTIVE (F(2,18) = 0.86, p = 0.441, two-way ANOVA; Fig 5A).

#### Mushroom spines

**Fig 6:**
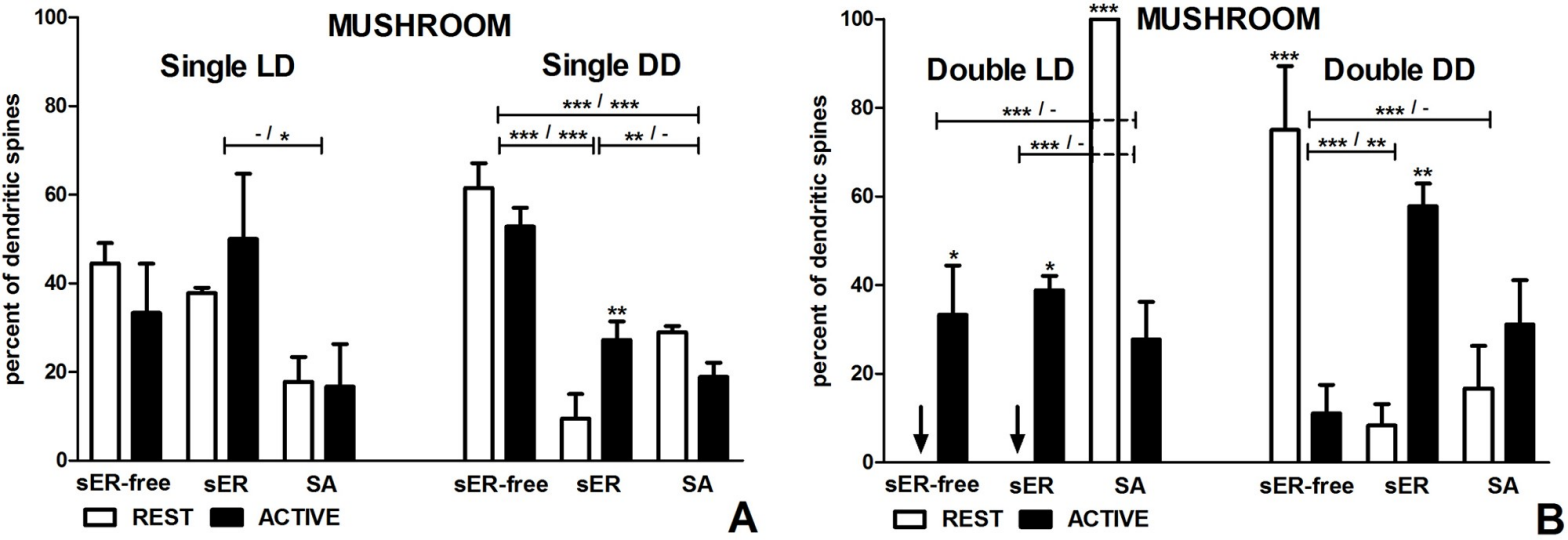
Content of mushroom spines in groups REST and ACTIVE under LD and DD conditions. **Content changes in single- and double-synapse spines are driven by both, circadian clock and light.** (A) Mushroom single-synapse spines. (B) Mushroom double-synapse spines. The graphs show means ± SEM (two-way ANOVA; *** p < 0.001, ** p < 0.01, * p < 0.05). Arrows indicate the absence of spines. The asterisks above the bars indicate significant differences in the percentage of spines with the same content between activity phases (REST vs ACTIVE), while the asterisks above lines show significant differences between spines with different contents for the corresponding REST / ACTIVE phase. sER-free–no membranous structures; sER–smooth endoplasmic reticulum; SA–spine apparatus.

In the LD group, there were no differences in mushroom single-synapse spines between the subgroups REST and ACTIVE, irrespective of the spine content (F(2,18) = 1.13, p = 0.346, two-way ANOVA; Figs 2 and 6A). However, mushroom single-synapse spines containing sER were twice more frequent than those containing SA in the subgroup ACTIVE (t = 3.022, p < 0.05; F(2,18) = 6.61, p = 0.007, two-way ANOVA; Fig 6A).

In the DD group, mushroom sER-free single-synapse spines were more frequent than mushroom single-synapse spines containing sER (six and two times in the subgroups REST and ACTIVE, respectively) and spines containing SA (twofold and almost threefold in the subgroups REST and ACTIVE, respectively) regardless of the activity phase (REST–sER: t = 9.969, p < 0.001; SA: t = 6.240, p < 0.001; ACTIVE–sER: t = 4.901, p < 0.001; SA: t = 6.499, p < 0.001; F(2,18) = 64.66, p < 0.0001, two-way ANOVA; Fig 6A). Moreover, mushroom single-synapse spines containing SA were threefold more numerous than mushroom single-synapse spines containing sER in the subgroup REST (t = 3.729, p < 0.01; F(2,18) = 64.66, p < 0.0001, two-way ANOVA; Fig 6A). The only activity associated difference concerned mushroom single-synapse spines containing sER which were almost threefold more frequent in the subgroup ACTIVE when compared with REST (t = 3.394, p < 0.01; F(2,18) = 9.02, p = 0.0019, two-way ANOVA; Fig 6A).

#### Intermediate spines

**Fig 7:**
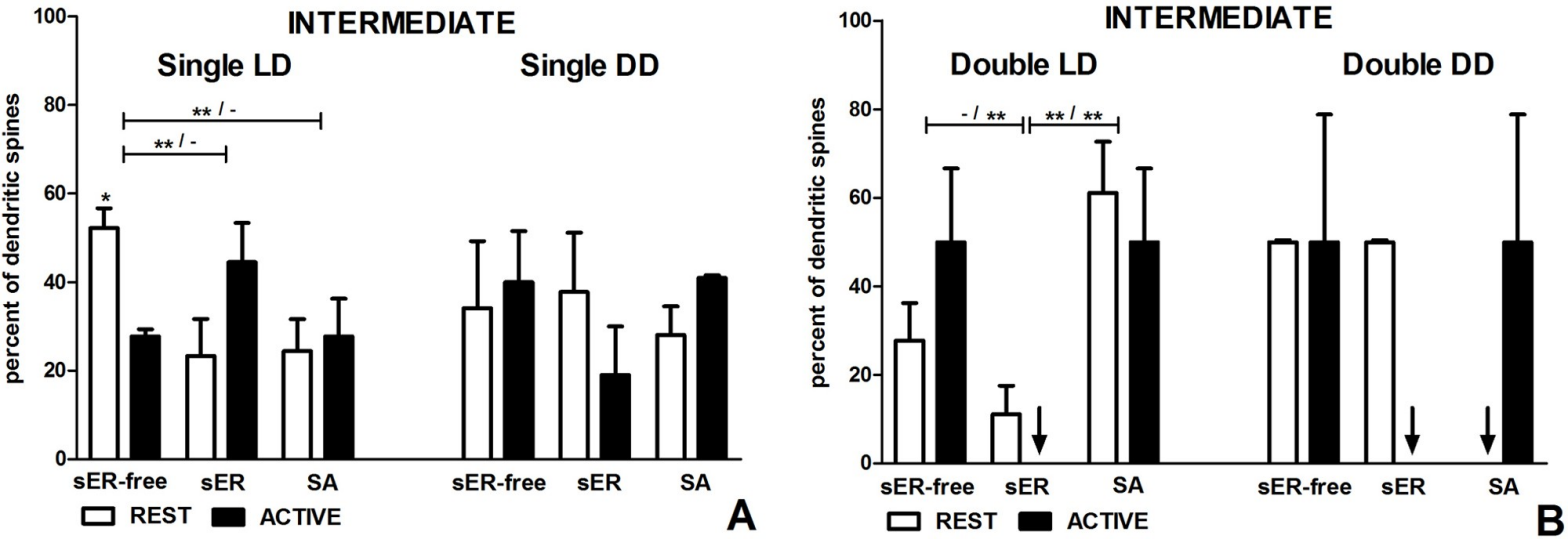
Content of intermediate spines in groups REST and ACTIVE under LD and DD conditions. **Content of single- and double-synapse spines does not change between activity phases except for sER-free single-synapse spines.** (A) Intermediate single-synapse spines. (B) Intermediate double-synapse spines. The graphs show means ± SEM (two-way ANOVA; *** p < 0.001, ** p < 0.01, * p < 0.05). Arrows indicate the absence of spines. The asterisks above the bars indicate significant differences in the percentage of spines with the same content between activity phases (REST vs ACTIVE), while the asterisks above lines show significant differences between spines with different contents for the corresponding REST / ACTIVE phase. sER-free–no membranous structures; sER–smooth endoplasmic reticulum; SA–spine apparatus.

In the LD group, the most numerous intermediate single-synapse spines were sER-free spines in the subgroup REST, being more frequent compared to intermediate single-synapse spines containing sER (t = 3.359, p < 0.01) and SA (t = 3.230, p < 0.01; F(2,18) = 7.12, p = 0.005, two-way ANOVA; Figs 2 and 7A). Intermediate sER-free single-synapse spines were also almost twice more frequent in the subgroup REST compared to ACTIVE (t = 2.841, p < 0.05; F(2,18) = 7.12, p = 0.005, two-way ANOVA; Fig 7A). However, there were no significant differences in intermediate single-synapse spines containing sER and containing SA between the subgroups REST and ACTIVE (sER: t = 2.454, p > 0.05; SA: t = 0.388, p > 0.05; F(1, 18) = 0.0000004, p = 0.999, two-way ANOVA; Fig 7A).

In the DD group, there were no differences in the content of intermediate single-synapse spines between the subgroups REST and ACTIVE (F(2,18) = 1.56, p = 0.237, two-way ANOVA; Fig 7A). No differences, either, were found between spines containing the particular type of membranous structures irrespective of the activity phases (F(2,18) = 0.45, p = 0.645, two-way ANOVA; Fig 7A).

### Shape to content relationship in double-synapse spines

#### Stubby spines

In the LD group, all stubby double-synapse spines in the subgroup REST contained sER, what resulted in significant differences in the frequency of stubby double-synapse spines containing sER compared to stubby sER-free double-synapse spines and stubby double-synapse spines containing SA (sER-free: t = 8.486, p < 0.001; SA: t = 8.486, p < 0.001; F(2,15) = 22.29, p < 0.0001, two-way ANOVA; Figs 2 and 4B). Consequently, stubby double-synapse spines containing sER were fourfold more numerous in the subgroup REST compared to ACTIVE (t = 6.804, p < 0.001; F(2,15) = 36.01, p < 0.0001, two-way ANOVA; Fig 4B). Additionally, stubby sER-free double-synapse spines were significantly more frequent in the subgroup ACTIVE compared to REST (t = 4.536, p < 0.01; F(2,15) = 36.01, p < 0.0001, two-way ANOVA; Fig 4B). However, there was no difference between the subgroups REST and ACTIVE in the percentage of stubby double-synapse spines containing SA (t = 2.268, p > 0.05; Fig 4B).

In the DD group, there were no differences in the percentage of stubby double-synapse spines between the subgroups REST and ACTIVE regardless of the spine content (F(2,12) = 1.131, p = 0.355, two-way ANOVA; Fig 4B). No differences, either, were found between spines with the particular content type irrespective of the activity phases (F(2,12) = 0.429, p = 0.660, two-way ANOVA; Fig 4B).

#### Thin spines

In the LD group, no significant differences were observed in the percentage of thin double-synapse spines between different activity phases and between spines with the particular content type (F(2,18) = 2.691, p = 0.055, two-way ANOVA; Figs 2 and 5B).

In the DD group, there were no thin sER-free double-synapse spines and thin double-synapse spines containing sER in the subgroups REST and ACTIVE, respectively (Fig 5B). Therefore, we observed significant differences in the percentage of thin sER-free double-synapse spines and thin double-synapse spines containing sER between the subgroups REST and ACTIVE (sER-free: t = 200, p < 0.001; sER: t = 200, p < 0.001; F(2,10) = 42860, p < 0.0001, two-way ANOVA; Fig 5B), as well as between thin sER-free double-synapse spines and thin double-synapse spines containing sER in the subgroups REST (t = 200, p < 0.001) and ACTIVE (t = 200, p < 0.001; F(2,10) = 14290, p < 0.0001, two-way ANOVA; Fig 5B). Moreover, thin double-synapse spines containing SA were more numerous than thin sER-free double-synapse spines and thin double-synapse spines containing sER in the subgroups REST and ACTIVE, respectively (sER-free: t = 223, p < 0.001, sER: t = 223, p < 0.001; F(2,10) = 14290, p < 0.0001, two-way ANOVA; Fig 5B).

#### Mushroom spines

In the LD group, all mushroom double-synapse spines were spines containing SA in the subgroup REST, while in the subgroup ACTIVE mushroom double-synapse spines were equally divided between all three content types (Figs 2 and 6B). Therefore, significant differences between the subgroups REST and ACTIVE were observed in each type of spines (sER-free: t = 3.097, p < 0.05, sER: t = 3.614, p < 0.05, SA: t = 6.712, p < 0.001; F(2,12) = 33.85, p < 0.0001, two-way ANOVA; Fig 6B). Additionally, mushroom double-synapse spines containing SA were more numerous than mushroom sER-free double-synapse spines and mushroom double-synapse spines containing sER in the subgroup REST (sER-free: t = 8.048, p < 0.001, SA: t = 8.048, p < 0.001; F(2,12) = 24.26, p < 0.0001, two-way ANOVA; Fig 6B).

In the DD group, the most numerous mushroom double-synapse spines were sER-free spines in the subgroup REST and they were nine- and fourfold more frequent compared to mushroom double-synapse spines containing sER and containing SA, respectively (sER: t = 5.379, p < 0.001; SA: t = 4.706, p < 0.001; F(2,15) = 25.06, p < 0.0001,two-way ANOVA; Fig 6B). Mushroom double-synapse spines containing sER prevailed in the subgroup ACTIVE and they were fivefold more numerous than mushroom sER-free double-synapse spines (t = 4.348, p < 0.01; F(2,15) = 25.06, p < 0.0001,two-way ANOVA; Fig 6B). Moreover, mushroom sER-free double-synapse spines and mushroom double-synapse spines containing sER were almost sevenfold more numerous in the subgroups REST and ACTIVE respectively, as compared with the other activity phase (sER-free: t = 5.511, p < 0.001, sER: t = 4.265, p < 0.01; F(2,15) = 25.06, p < 0.0001,two-way ANOVA; Fig 6B).

#### Intermediate spines

In the LD group, intermediate double-synapse spines containing sER were absent in the subgroup ACTIVE, which resulted in significant differences between these spines and the other types of intermediate double-synapse spines (sER-free: t = 3.530, p < 0.01; SA: t = 3.530, p < 0.01; F(2,18) = 12.92, p = 0.0003, two-way ANOVA; Figs 2 and 7B). Intermediate double-synapse spines containing SA were fivefold more numerous than intermediate double-synapse spines containing sER in the subgroup REST (t = 3.530, p < 0.01; F(2,18) = 12.92, p = 0.0003, two-way ANOVA; Fig 7B). There were no differences in the percentage of all intermediate double-synapse spine types between the subgroups REST and ACTIVE (F(2,18) = 1.85, p = 0.187, two-way ANOVA; Fig 7B).

In the DD group, there were no intermediate double-synapse spines containing sER and containing SA in the subgroups ACTIVE and REST, respectively. Despite that, no significant differences between the subgroups REST and ACTIVE (F(2,9) = 2.70, p = 0.121, two-way ANOVA; Fig 7B) were found due to very high standard deviations in the subgroup ACTIVE.

## Discussion

The overwhelming number of synapses localized on dendritic spines, as compared to synapses in the other locations (dendritic shaft, soma), underscores their importance for synaptic transmission. Diurnal structural changes of dendritic spines seem to be a natural consequence of their high motility [46,47]. Dendritic spines are able to change their shapes very quickly by activity-dependent and independent mechanisms [48–51]. Morphological modifications of spines seem to be at least as important as quantitative changes and physiological alterations are better reflected in morphology than in density of spines [10,52].

Single-synapse spines are the overwhelming population of spines in the somatosensory cortex, while double-synapse spines (with one excitatory and one inhibitory synapse) constitute only about 10% of spines [53], however, they are the main target of experience-dependent plasticity in this region [42,43,54]. The excitatory synapses are usually located on the heads of double-synapse spines, while the inhibitory synapses are placed on the spine necks [53]. The arrangement of synapses on double-synapse spine allows for easy reduction/regulation of the excitatory synapse response transmitted through the spine neck to the dendritic shaft [55,56] and makes double-synapse spines important excitation modifying devices.

The results of this study demonstrate for the first time the circadian rhythmicity of spine shape changes in mammalian brain. Limitation of this study is the cross-sectional analysis which provides weaker arguments supporting causal relationships. However, in all chronobiological studies which require collection of brain tissue samples it is the only possible type of analysis.

Layer IV of mouse somatosensory cortex contains whisker representations in the form of distinct cytoarchitectonic structures, barrels [57]. The locomotor activity of animals is accompanied by stimulation of whiskers, hence its changes directly influence synapses in the barrels. As the intensity of locomotor activity cyclically changes during 24h period under the influence of the circadian clock, synapses are cyclically modified. An important source of synaptic changes in the LD regime is also light which entrains the circadian clock but has not direct effect on whiskers. The light-stimulated pathway via the retinohypothalamic tract leads to the suprachiasmatic nucleus, the mammalian clock [58–60], and to brain regions associated with attention and vigilance [61,62] and may indirectly affect neuronal changes in the somatosensory cortex. Comparison of synaptic modifications in LD and DD conditions allows to determine whether cyclic plastic changes are driven by the circadian clock, or by light. Changes between the activity phases observed in constant darkness (DD) are driven exclusively by the biological clock and changes observed under identical experimental conditions in LD but not in DD can be attributed to the influence of light. Table 1 summarizes the effects of the circadian clock and light on the morphology of dendritic spines.

**Table 1:** Influence of light and the circadian clock on morphology of single- and double-synapse spines with different content in mouse barrel cortex.

	Single-synapse spines	Double-synapse spines
**Effect of light**	Increase in the number of: stubby spines containing sER*, mushroom spines (especially containing sER)*, intermediate sER-free spines	Increase in the number of: stubby spines containing sER, thin spines (especially sER-free)*, mushroom spines containing SA, thin/mushroom spines
	Decrease in the number of: stubby sER-free spines	Decrease in the number of: stubby sER-free spines, mushroom spines (especially sER-free)
**Effect of circadian clock/** **locomotor activity** **(endogenous effect)**	Increase in the number of: stubby spines containing sER*, thin spines containing sER, mushroom spines (especially containing sER)*	Increase in the number of: thin sER-free spines*, mushroom spines containing sER
	Decrease in the number of: stubby spines containing SA	Decrease in the number of: thin spines containing sER, mushroom sER-free spines

sER, smooth endoplasmic reticulum; SA, spine apparatus.

*Spines influenced by both factors.

### Circadian plasticity of single synapse-spines

Under both, LD and DD conditions, the number of single-synapse spines increases during the day/subjective day, what means the observed changes are endogenous [41]. Comparing morphological spine changes under light/dark regime and in constant darkness we found that the shape modifications of single-synapse spines are generally driven by the circadian clock and only in case of mushroom spines they are influenced by light: in LD mainly mushroom single-synapse spines are formed and this process is driven by light. Interestingly, increase in the number of stubby and mushroom single-synapse spines containing sER is induced by locomotor activity as well as by light. This suggests that an increase in transportation of sER from dendritic shafts to spines is independent of the stimulus type.

We found that clock/activity of the animals in DD and light in LD promoted cyclic formation of mushroom single-synapse spines. Considering a continuum of spine stability based on spine shapes (stubby–thin–mushroom), this result seems to be especially interesting because the mushroom spines regarded as very stable [5,10]. However, results reported by some other authors suggest that thin, stubby and mushroom spines have the same longevity [63]. Since the observed changes are cyclic, it seems that the formation and degradation of mushroom single-synapse spines cannot be explained exclusively by formation of new ‘memory spines’.

Stubby single-synapse spines showed the greatest diurnal and circadian variability of their content, while thin single-synapse spines remained the most stable, as far as their content was concerned. The source of content alterations in stubby single-synapse spines seems to be more diverse than in case of the other single-synapse spines: in some spines they were driven by the circadian clock and in the others by light. Percentage differences of thin and mushroom single-synapse spines with the particular content type between the activity phases were observed only in DD, indicating that the changes were exclusively endogenous. Under both investigated conditions spines containing SA were the least numerous among mushroom single-synapse spines. This is in accordance with our expectations, because mushroom spines containing SA are regarded to be most mature and stable [2,35], therefore their number should not significantly change.

### Circadian plasticity of double-synapse spines

In contrast to single-synapse spines, the shape changes of double-synapse spines are driven exclusively by light. The variations in the content of these spines were also different from those observed in single-synapse spines. In thin double-synapse spines they were endogenous, while in stubby double-synapse spines they were driven by light and in mushroom double-synapse spines by both factors. Under LD conditions we observed an unusual situation: during the day all mushroom double-synapse spines contained SA and all stubby double-synapse spines contained sER, whereas in the night the fractions of all content types of mushroom and stubby double-synapse spines were similar. Generally, spines containing SA are considered to be the least variable, regardless of shape [35]. This rule, however, does not seem to apply to mushroom double-synapse spines, although they are regarded as most mature and stable spines [2,64].

The total number of double-synapse spines increases during the night/subjective night under both LD and DD conditions and similarly to single-synapse spines, the changes are endogenous [41]. The newly formed double-synapse spines are either sER-free or contain sER. It means that the nascent double-synapse spines are immature spines, as far as their content is concerned. Newly formed single-synapse spines are thought to contain no organelles [35]. However, as observed during the learning processes, double-synapse spines are formed by addition of inhibitory synapse to a large and mature single-synapse mushroom spine containing SA [42,45,53]. This difference suggests that the neuroplasticity observed in the circadian cycles is exceptional due to the rhythmic nature of the process and short time available for changes. The light induces transformation of existing double-synapse spines towards the immature form (sER-free or containing sER), while in darkness all types of double-synapse spines can be formed with equal probability. However, there seems to be no preference for any shape of single-synapse spines to attach inhibitory synapses and to convert into double-synapse spines.

### Circadian trends of spine transformations and maturation

Intermediate single-synapse spines were twice as numerous as intermediate double-synapse spines. It could suggest a longer time needed to achieve a specific shape by single-synapse spines than by double-synapse spines. The other possibility is that single-synapse spines are generally less stable than double-synapse spines. On the other hand, single-synapse spines are formed de novo, while formation of new double-synapse spines requires existence of single-synapse spines [42,43].

The negligible number of stubby/thin and large number of thin/mushroom single- and double-synapse spines during the light phase (under LD) suggests that light preferentially inhibits transformation of thin spines into stubby spines. Moreover, the number of mushroom single-synapse spines increases in the light phase confirming that the light promotes modifications of spines towards mature ones. Although the number of mushroom double-synapse spines did not increase during the light phase, all mushroom double-synapse spines were mature (contained SA).

### Influence of stress hormones on dendritic spine changes

It seems that an additional factor that might affect the formation of dendritic spines or their morphology is the cyclic secretion of glucocorticoids by the adrenal cortex [40,65], subordinated to the hierarchical system referred to as the hypothalamic-pituitary-adrenal (HPA) axis. Glucocorticoids control various animal functions related to physiology and metabolism, and are particularly involved in controlling stress response [66–68]. The central clock in the suprachiasmatic nucleus is responsible for generating circadian rhythms in peripheral clocks located in other tissues of the body [69–71]. The peripheral adrenal clock, although coordinated by the central clock and unable to generate its own rhythm, might influence the other peripheral oscillators by the rhythmic secretion of glucocorticoids [66,68,72].

In nocturnal animals, the peak of glucocorticoid secretion occurs at the beginning of night [73,74]. Increase in the level of these hormones leads to an enhanced synapse turnover, including both, formation and elimination of synapses [75]. The rhythmic oscillations of glucocorticoids appear to be important for formation or maintenance of nascent spines after learning [65]. The number of spines with large heads (large spines) in hippocampal CA1 field neurons in increase at the beginning of the active phase under LD [40]. In the somatosensory cortex, we observed similar rhythmics of mushroom double-synapse spines, while the number of mushroom single-synapse spines did not change between day and night in LD. The changes of mushroom single-synapse spines between the two activity phases were found only in DD. However, it is difficult to exclude that the changes observed in this study might partly result from cyclic glucocorticoid secretion.

### Functional implications

The observation of mature spine (thin and mushroom) changes leads to an interesting conclusion: thin spines probably act as ‘learning spines’ [2], but only some mushroom spines are ‘write-protected’ spines [2] in the circadian cycle. The cyclic formation and degradation of mushroom single-synapse spines cannot be explained exclusively by formation of new ‘memory spines’. Mushroom spines are usually big spines with large heads, characterized by the presence of large, very effective and strong synapses containing numerous glutamate receptors. In the active phase, there were fewer single-synapse spines comparing with the rest phase [41], so it seems that an increase in the strength of synapses resulting from change of the spine shape into mushroom-shaped might be a more efficient and faster way to achieve enhanced neurotransmission than a change of spine number. The light is a stressful condition for nocturnal rodents and requires not only more synapses to increase the sensitivity to weak stimuli [41], but also increased strength of excitatory synapses to enhance the effectiveness of neurotransmission. Similarly, the presence of only double-synapse spines containing SA in the light phase indicates more effective regulation of calcium [25,76,77] as well as increased protein production [3] in the spine, what can also be related to the potentiation of synapses.

## Conclusions

Results obtained in the present study demonstrate that in addition to quantitative changes of spines [41], they also undergo qualitative modifications within 24 h period. Shape alterations of single-synapse spines are driven by the circadian clock and modified by light, while changes of double-synapse spines are under the influence of light. Generally, during the light phase large, mature spines are formed preferentially. It seems to be related more to the increase in the strength of synapses accompanying large spines than to the significance of the mushroom spines for memory storage.

## Supporting information

S1 Table Percentages of single-synapse spines with different shapes in the two phases of the circadian cycle. Table shows mean ± SD.
(DOCX)

S2 Table Percentages of double-synapse spines with different shapes in the two phases of the circadian cycle. Table shows mean ± SD.
(DOCX)
