# Supplementary material for: Circadian clock regulates the shape and content of dendritic spines in mouse barrel cortex
Source: PLoS One. 2019 Nov 15;14(11):e0225394. doi: 10.1371/journal.pone.0225394 (PMC6857954; doi:10.1371/journal.pone.0225394)
Supplement: S1 Table — Table shows mean ± SD. (DOCX) [file pone.0225394.s001.docx]

| **SINGLE-SYNAPSE SPINES** | | | |
| --- | --- | --- | --- |
|  | **Shape** | **REST** | **ACTIVE** |
| **LD** | Stubby | Total: 9.39 ± 9.11%  sER-free: absent  sER: 50.00 ± 0.00%  SA: 50.00 ± 0.00% | Total: 13.35 ± 3.88%  sER-free: 33.33 ± 16.67%  sER: absent  SA: 66.67 ± 16.67% |
|  | Thin | Total: 15.76 ± 14.12%  sER-free: 41.67 ± 8.33%  sER: 12.50 ± 12.50%  SA: 45.84 ± 20.84% | Total: 18.90 ± 12.76%  sER-free: 41.67 ± 22.05%  sER: absent  SA: 58.33 ± 22.05% |
|  | Mushroom | Total: 45.30 ± 0.26%  sER-free: 44.44 ± 8.01%  sER: 37.78 ± 2.22%  SA: 17.78 ± 9.69% | Total: 38.02 ± 19.39%  sER-free: 33.33 ± 19.25%  sER: 50.00 ± 25.46%  SA: 16.67 ± 16.67% |
|  | Intermediate | Stubby/thin: absent  Thin/mushroom: 29.54 ± 6.01%  sER-free: 52.22 ± 7.78%  sER: 23.33 ± 14.53%  SA: 24.44 ± 12.37% | Stubby/thin: 5.16 ± 4.51%  Thin/mushroom: 24.57 ± 2.94%  sER-free: 27.78 ± 2.78%  sER: 44.44 ± 15.47%  SA: 27.78 ± 14.70% |
| **DD** | Stubby | Total: 26.35 ± 2.40%  sER-free: 46.67 ± 3.33%  sER: absent  SA: 53.33 ± 3.33% | Total: 17.72 ± 6.69%  sER-free: 45.56 ± 13.65%  sER: 34.44 ± 8.68%  SA: 20.00 ± 20.20% |
|  | Thin | Total: 10.16 ± 3.84%  sER-free: 50.00 ± 28.87%  sER: absent  SA: 50.00 ± 28.87% | Total: 5.50 ± 1.46%  sER-free: 16.67 ± 16.67%  sER: 50.00 ± 28.87%  SA: 33.33 ± 33.33% |
|  | Mushroom | Total: 27.14 ± 5.96%  sER-free: 61.51 ± 9.63%  sER: 9.52 ± 9.52%  SA: 28.97 ± 2.41% | Total: 51.91 ± 1.80%  sER-free: 52.78 ± 7.35%  sER: 27.22 ± 7.22%  SA: 18.89 ± 5.56% |
|  | Intermediate | Stubby/thin: 6.19 ± 1.26  Thin/mushroom: 30.16 ± 2.75%  sER-free: 34.13 ± 26.24%  sER: 37.78 ± 23.20%  SA: 28.09 ± 11.08% | Stubby/thin: 2.90 ± 5.02%  Thin/mushroom: 21.97 ± 11.54%  sER-free: 40.00 ± 20.00%  sER: 19.05 ± 19.05%  SA: 40.95 ± 0.95% |
