# Supplementary material for: Circadian clock regulates the shape and content of dendritic spines in mouse barrel cortex
Source: PLoS One. 2019 Nov 15;14(11):e0225394. doi: 10.1371/journal.pone.0225394 (PMC6857954; doi:10.1371/journal.pone.0225394)
Supplement: S2 Table — Table shows mean ± SD. (DOCX) [file pone.0225394.s002.docx]

| **DOUBLE-SYNAPSE SPINES** | | | |
| --- | --- | --- | --- |
|  | **Shape** | **REST** | **ACTIVE** |
| **LD** | Stubby | Total: 14.07 ± 7.07%  sER-free: absent  sER: 100 ± 0.0%  SA: absent | Total: 35.65 ± 11.68%  sER-free: 50.00 ± 25.00%  sER: 25.00 ± 14.43%  SA: 25.00 ± 14.43% |
|  | Thin | Total: 36.48 ± 5.88%  sER-free: 33.33 ± 16.67%  sER: 50.00 ± 28.87%  SA: 16.67 ± 16.67% | Total: 16.20 ± 4.42%  sER-free: 33.33 ± 33.33%  sER: 16.67 ± 16.67%  SA: 50.00 ± 28.87% |
|  | Mushroom | Total: 16.67 ± 16.67%  sER-free: absent  sER: absent  SA: 100.00 ± 0.00% | Total: 31.94 ± 3.67%  sER-free: 33.33 ± 19.25%  sER: 38.89 ± 5.56%  SA: 27.78 ± 14.70% |
|  | Intermediate | Stubby/thin: absent  Thin/mushroom: 32.78 ± 4.34%  sER-free: 27.78 ± 14.70%  sER: 11.11 ± 11.11%  SA: 61.11 ± 20.03% | Stubby/thin: 4.17 ± 4.17%  Thin/mushroom: 12.04 ± 0.46%  sER-free: 50.00 ± 28.87%  sER: absent  SA: 50.00 ± 28.87% |
| **DD** | Stubby | Total: 40.66 ± 2.20%  sER-free: 43.34 ± 23.34%  sER: 36.67 ± 3.33%  SA: 20.00 ± 20.00% | Total: 34.72 ± 19.30%  sER-free: 35.42 ± 2.08%  sER: 25.00 ± 25.00%  SA: 39.59 ± 27.09% |
|  | Thin | Total: 14.29 ± 14.29%  sER-free: absent  sER: 50.00 ± 0.0%  SA: 50.00 ± 0.0% | Total: 11.11 ± 11.11%  sER-free: 50.00 ± 0.0%  sER: absent  SA: 50.00 ± 0.0% |
|  | Mushroom | Total: 37.36 ± 8.79%  sER-free: 75.00 ± 25.00%  sER: 8.33 ± 8.33%  SA: 16.67 ± 16.67% | Total: 45.83 ± 11.02%  sER-free: 11.11 ± 11.11%  sER: 57.78 ± 8.89%  SA: 31.11 ± 17.36% |
|  | Intermediate | Stubby/thin: 7.69 ± 7.69%  Thin/mushroom: absent  sER-free: 50.00 ± 0.00%  sER: 50.00 ± 0.00%  SA: absent | Stubby/thin: absent  Thin/mushroom: 8.33 ± 8.34%  sER-free: 50.00 ± 50.00%  sER: absent  SA: 50.00 ± 50.00% |
